# Unravelling the Role of Metallothionein on Development, Reproduction and Detoxification in the Wall Lizard *Podarcis sicula*

**DOI:** 10.3390/ijms18071569

**Published:** 2017-07-19

**Authors:** Rosaria Scudiero, Mariailaria Verderame, Chiara Maria Motta, Palma Simoniello

**Affiliations:** 1 Department of Biology, University Federico II, Via Mezzocannone 8, 80134 Napoli, Italy; ilaria.verderame@unina.it (M.V.); chiaramaria.motta@unina.it (C.M.M.); 2 Department of Sciences and Technology, University Parthenope, Centro Direzionale, Isola C4, 80143 Napoli, Italy; palma.simoniello@uniparthenope.it

**Keywords:** *Podarcis sicula* metallothionein, gene expression, in situ hybridisation, embryonic development, cadmium, oogenesis, spermatogenesis

## Abstract

Metallothioneins (MTs) are an evolutionary conserved multigene family of proteins whose role was initially identified in binding essential metals. The physiological role of MT, however, has been revealed to be more complex than expected, since not only are MTs able to bind to toxic heavy metals, but many isoforms have shown specialized and alternative functions. Within this uncertainty, the information available on MTs in non-mammalian vertebrates, particularly in neglected tetrapods such as the reptiles, is even more scant. In this review, we provide a summary of the current understanding on metallothionein presence and function in the oviparous lizard *Podarcis sicula*, highlighting the results obtained by studying *MT* gene expression in most representative adult and embryonic tissues. The results demonstrate that in adults, cadmium induces MT transcription in a dose- and tissue-specific manner. Thus, the MT mRNAs appear, at least in some cases, to be an unsuitable tool for detecting environmental ion contamination. In early embryos, maternal RNAs sustain developmental needs for MT protein until organogenesis is well on its way. At this time, transcription starts, but again in a tissue- and organ-specific manner, suggesting an involvement in alternative roles. In conclusion, the spatiotemporal distribution of transcripts in adults and embryos definitively confirms that MT has deserved the title of elusive protein.

## 1. Introduction

Since its discovery in 1957 from horse kidney as a cadmium-binding protein [1], metallothionein (MT) functions have been associated with metal micronutrients homeostasis and heavy metals detoxification [2]. Despite many studies aimed at validating the use of MT as a good biomarker of heavy metal contamination, increasing evidence demonstrates the involvement of this protein in many normal or pathological cellular processes, so that MT has deserved the title of elusive protein [3,4].

Found in all the eukaryotic taxa [5], MT has been extensively studied in invertebrates and vertebrates, both at the evolutionary and structural levels [6,7,8,9,10]. Among vertebrates, MT studies are particularly numerous on mammals, while within the lower vertebrates, the MTs have been studied in greater detail only in fish, in relation to their detoxifying capacity and the significant metal pollution of the aquatic environment [6,11,12].

Metallothioneins belong to a multigenic family. In mammals, four tandemly clustered genes (*MT1* to *MT4*) have been identified, with *MT1* and *MT2* being present in almost all tissues, and *MT3* and *MT4* being more abundant in neuronal and epithelial cells, respectively [13,14]. In addition, *MT1* has undergone further duplication events that have resulted in many duplicate isoforms (13 in humans, from *MT1a* to *MT1m*) [15].

At first, MTs were considered structurally conserved among vertebrates; recent studies however have demonstrated a greater complexity than expected, resulting in a certain confusion in the current nomenclature of the different MT types [16,17]. Indeed, mammalian MT4 and avian MT1 are the most ancestral isoforms in tetrapods, the MT1 and MT2 isoforms do not have a monophyletic origin, and finally, mammalian MT3 clusters form a sister group of the amphibian MT clade [18]. In the latter, an atypical MT has recently been identified, characterised by a lower number of cysteine residues (17 instead of 20) and by two Cys–Cys–Cys motifs [19].

**Figure 1:**
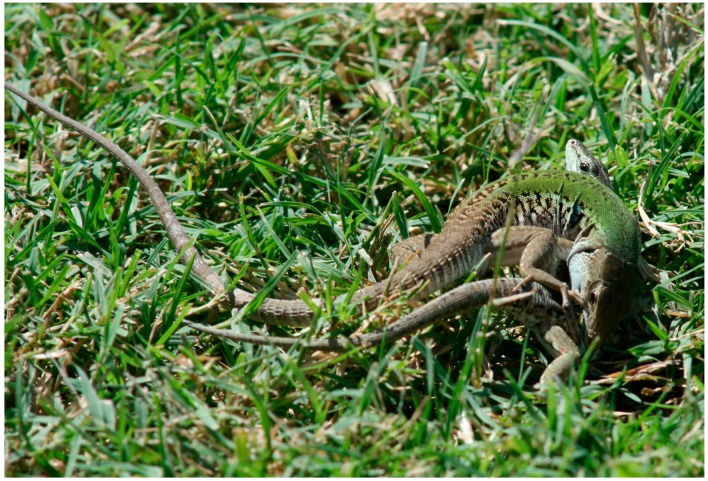
Adult specimens of *Podarcis sicula* during copulation.

Although a plethora of studies have attempted to clarify MT evolution in vertebrates [15,16,17,18,19,20,21], little attention has been dedicated to the MTs in non-mammalian vertebrates, particularly in neglected tetrapods such as reptiles. Therefore, to shed some light on the presence and function of this protein in adult and embryos of an amniotic egg-laying tetrapod, we have undertaken studies using as experimental model the Italian wall lizard *Podarcis sicula* (Figure 1).

Here, we provide a summary of the current understanding on MTs’ presence and function, highlighting the results obtained by studying its expression by molecular and cytological approaches.

## 2. *P. sicula* Metallothionein

**Figure 2:**
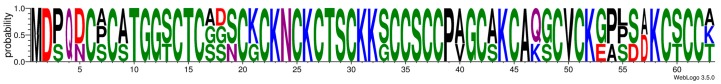
Graphical representation of amino acidic sequence similarity among rat, chicken, and lizard metallothioneins (MTs).

The cDNA encoding the *P. sicula* MT was firstly isolated from the liver (GenBank accession number AJ609541) [22]. The protein (UniProt Q708T3) is made of 63 amino acids with 20 cysteines arranged in a fashion typical of vertebrates MT1 and MT2; *P. sicula* MT shows about 80% identity and 85% similarity with both mammalian and avian MTs (Figure 2). To date, no other isoforms have been found, not in other tissues (including the brain), or after cadmium induction [20,23].

## 3. Metallothionein in *P. sicula* Adult Tissues

Gene expression analyses performed on tissues of *P. sicula* adult specimens collected in rural areas show that MT transcripts are present in all of the five tissues examined (Table 1). Interestingly, the highest amount of transcript is found in the brain, followed by the two detoxifying organs, liver and kidney, then the ovary and the gut (brain > liver > kidney > ovary > gut) [23]. In this regard, we should point out that there is only one MT isoform in lizard brain; therefore it can be postulated that its abundance would permit those functions that in mammalian brains are accomplished by MT1/MT2 and MT3.

To test short- and long-term Cd effects on MT expression, adult lizards were exposed to acute or chronic treatments [23,24,25,26]. In the former case, a single intra-peritoneal Cd administration (2 µg/g body mass) increases MT transcript content (Table 1), in concomitance with the accumulation of Cd ions [23]. The only exception is represented by the brain, in which the MT mRNA content remains unchanged. We have already demonstrated that both intra-peritoneally and dietary administered cadmium crosses the blood-brain barrier and accumulates in the brain [23]. Hence, it is evident that the lack of induction is not due to the absence of Cd ions. However, since the analysis is carried out on the whole organ, it cannot be excluded that up and down regulations occurred in different brain districts, leaving the total measured concentration unaltered. This possibility is now under investigation by cytological analyses.

In the liver, a threefold increase in both MT transcript and protein is observed (Table 1); however, this Cd-induction does not alter MT mRNA localisation. MT transcripts in fact are detected exclusively in the large Kupffer cells and in monocytes in vessels, whereas the hepatocytes remain completely unstained, both under natural conditions and after Cd-treatment [26].

In the ovary, a single intra-peritoneal Cd-treatment gives rise to a threefold increase in MT transcript content after two days from administration. In this case, the cytological data demonstrate that transcript localisation significantly changes, moving from small follicular stem cells to large nurse cells [27].

The response to oral cadmium uptake is also different in the various tissues examined. The chronic dietary treatment (1 µg/g body mass, every second day, for 60 days) induces *MT* gene expression in the gut, kidney, and ovary, whereas it is ineffective in the liver and brain (Table 1). Possibly, the high constitutive amount of MT transcript present in these organs blocks any further increase of *MT* gene transcription.

Taken together, data indicate that under these experimental conditions, in *P. sicula* the relationship between Cd accumulation and MT transcript induction/concentration is not always predictable, depending on the organ and/or on the exposure route. The conclusion, therefore, is that monitoring MT mRNA is not necessarily a suitable tool for detecting environmental cadmium contamination. This conclusion is reinforced by the observation, in invertebrates and vertebrates, that the translational inhibition of MT mRNA transcripts occurs. Determining in parallel the changes in MT protein concentration might partly prevent misinterpretations in monitoring studies.

## 4. Metallothionein in *P. sicula* Reproductive Organs

### 4.1. Metallothionein in the Ovary

*P. sicula* is an oviparous species, characterised by a seasonal reproductive cycle with low gonadal activity in autumn-winter and maximal gonadal activity in spring-summer, coinciding, in females, with two or three ovulatory waves [28]. In this period, due to the rising temperature, typical morpho-physiological modifications occur in the ovary; these include the increase in the number and size of follicles, and the onset of vitellogenesis. At the end of the ovulatory period, the ovary enters a fall stasis until November, when a temporary recrudescence occurs. No vitellogenic follicles are produced, and the ovary enters a new stasis that lasts until the next spring [29].

Beside yolk, during oogenesis the oocytes store RNAs and organelles [30], and micronutrients such as copper and zinc [22] to support the early stages of embryo development. The rate of metals uptake, in particular, is clearly synchronized throughout the stages of maturation, since their increases correlate closely; at the end of oocyte maturation, the copper and zinc content in *Podarcis* eggs is similar, suggesting comparable requirements for both metals during embryogenesis [22].

**Figure 3:**
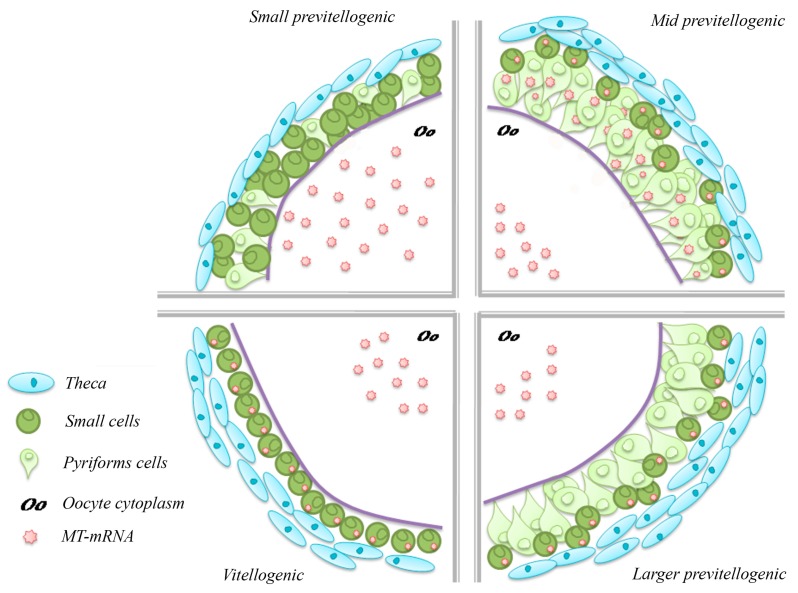
Schematic representation of the MT mRNA localisation in *P. sicula* follicles during oogenesis.

Interestingly enough, we found that in *P. sicula* oocytes and eggs both zinc and copper ions are associated with high molecular mass metal-binding proteins, whilst no metal is bound to low molecular mass proteins. The MTs are therefore absent throughout the ovary, in germ cells as in somatic follicle cells. However, MT transcripts are present: Northern blot analysis in fact demonstrates that they are expressed constitutively in the ovary, in all periods of the ovarian cycle, and that they accumulate during the reproductive period in ovaries containing large vitellogenic follicles approaching ovulation [22]. By using the in situ hybridisation technique, we have clarified where the MT mRNA is localised. In small previtellogenic follicles (<150 µm diameter), it localises only in the oocyte’s cytoplasm and not in the small stem cells forming the follicular epithelium. Later, in mid previtellogenesis (follicles 400–1400 µm diameter), the small stem cells, and to a lesser extent the differentiated pyriform cells, contain the MT transcripts. In even larger previtellogenic follicles (1.5–2 mm), the MT mRNA are localised in the small cells, and to a lesser extent in the regressing pyriform cells. In vitellogenic follicles (>2 mm diameter), pyriform cells are no longer present, and all the MT mRNA is localised in the cytoplasm of the small cells [27,31]. In the oocyte cytoplasm, the hybridisation signal is undetectable, indicating at first that no MT mRNA is present. However, the oocyte volume is very large, and the transcripts are dispersed and probably masked by the large yolk platelets (Figure 3). Their presence in fact is confirmed by molecular techniques [22]. The origin of the oocyte transcripts is also intriguing. The oocyte nucleus is silent since the early diplotene stage; however, the large pyriform cells synthesize RNAs, organelles, and cytoplasm that transfer into the oocyte cytoplasm via intercellular bridges at the end of previtellogenesis, before regressing [30].

The absence of protein indicates that the egg transcripts in *Podarcis* are stored to meet the future needs of the growing embryo. In effect, the MT mRNAs are still present in the discoblastulae [31]. Though in other species translation is resumed soon after fertilization [32], an asynchronous presence of transcripts and protein is rather common, and described, for example, in the embryos of the Roman snail *Helix pomatia* [33].

The induction of MT synthesis in ovaries following in vivo exposure to cadmium indicates that the translation of blocked MT mRNA can be triggered if an anomalous increase in metal content occurs [22]. This event is accompanied not only by an expected increase in MT expression (to restore the transcript pool), but, more significantly, by a change in transcript localisation, from small stem cells to differentiated pyriform cells [27]. The metal therefore seems to induce changes also in the function of the ovarian MTs.

### 4.2. Metallothionein in the Testis

The presence of MT in the testis of vertebrates has been a matter of debate for a long time, and for many years authors have reported a substantial lack of the protein [34,35]. Today, both MT expression and synthesis in testis has been widely demonstrated, even though cell localisation is still contradictory [36,37,38,39,40]. Evidence exists on MT localisation in both somatic and germ cells, but in line with what occurs in the ovary, the localisation changes over time, probably depending on the state of the gonad. In mouse and rat testes, high levels of MT transcripts are not always accompanied to MT protein co-localisation [41], as demonstrated for the *Podarcis* ovary.

Due to the lack of information, we decided to investigate the presence and the localisation of MT transcript and protein in the testis of *P. sicula*, in two representative phases of the annual reproductive cycle, to highlight the role of this protein during spermatogenesis. The *P. sicula* testis shows a tubular structure, like mammals. During the reproductive period, in spring-summer, the seminiferous epithelium contains germ cells in all of the stages of maturation, from spermatogonia to spermatozoa, and many sperms fill the lumen of the tubules. This period is followed by: (1) a late-summer stasis, characterised by the block of spermatogenesis; (2) an autumnal resumption, favoured by the increase in circulating testosterone, with the renewal of a defective spermatogenesis without spermiation; (3) a winter stasis; (4) an early spring resumption [42,43].

We performed in situ hybridisation and immunocytochemical analyses on *P. sicula* testis in the reproductive period and autumnal resumption. The results demonstrate the presence of MT transcript and protein in testicular cells; however, MT expression and synthesis are different in the two phases of the reproductive cycle [44].

**Figure 4:**
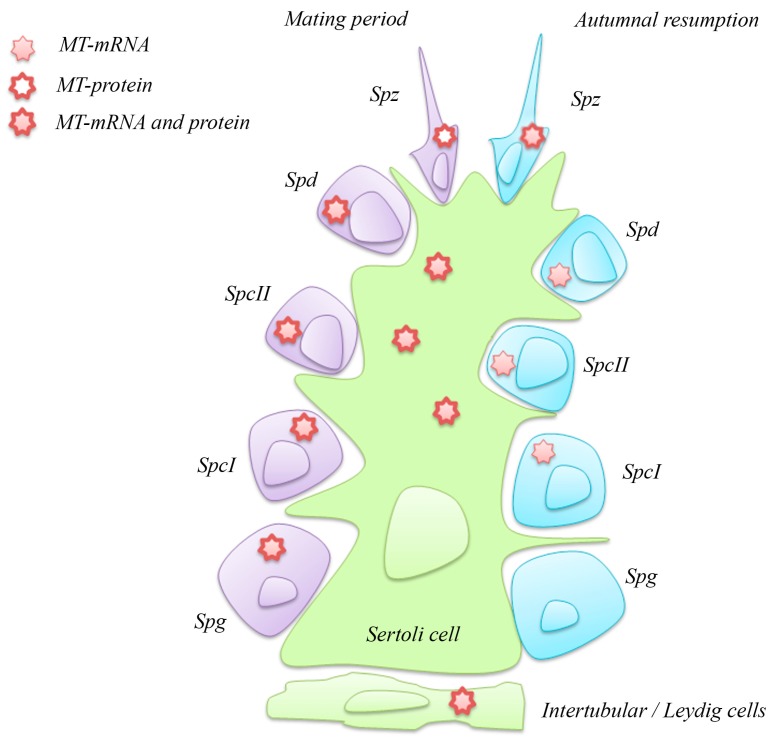
Schematic representation of MT mRNA and protein localisation in *P. sicula* testis during two stages of reproductive cycle. Spg, spermatogonia; SpcI, primary spermatocytes; SpcII, secondary spermatocytes; Spd, spermatids; Spz, spermatozoa.

In the mating period, spermatogonia, primary and secondary spermatocytes, and spermatids contain both MT transcript and protein, whereas spermatozoa show only the protein. This finding suggests that the MTs are translated in the earlier stages and the protein is stored, at least partly, for spermatozoa (Figure 4).

During the autumnal resumption, MT mRNA is present in primary and secondary spermatocytes, spermatids, and spermatozoa, but not in spermatogonia; conversely, the protein is evident only in spermatozoa (Figure 4). The spermatogonia are in a resting phase, the recruitment of new gametes being unnecessary; the spermatocytes and spermatids, probably left from the previous spermatogenetic wave, are also quiescent but maintain their MT mRNA untranslated after having depleted the MT protein store. The spermatozoa, almost certainly left from the past reproductive episode, maintain both transcripts and protein, a condition recalling that of summer spermatids. It would be interesting to analyse the ultrastructure of these cells to demonstrate their true maturation stage.

What is clear from these data is that many regulatory signals exist between transcription and the production of a functional protein, and that they can be conveniently studied in *Podarcis*, an animal model possessing a single MT protein isoform.

In Sertoli and intertubular cells, including the Leydig cells, MT transcript and protein are always present (Figure 4), a fact not surprising when it is considered that these two cell types are involved in the endocrine control of reproduction.

The presence of the MT protein in all spermatogenetic stages during the reproductive period may indicate a key role of MT in germ cells’ maturation, in particular in zinc distribution in the seminiferous epithelium. It is known that zinc ions, interacting with spermatozoa, are involved in sperm motility and viability, and the deficiency of this micronutrient leads to the atrophy of germ cells and a failure of sperm spawning [45,46]. In addition, a reversible metal exchange between MT and the zinc finger motifs of the estrogen receptors has been demonstrated; hence, the presence of MT in all spermatogenic cells during the mating period may ensure the proper zinc availability required for the correct functionality of the estrogen receptors [47].

## 5. Metallothionein in *P. sicula* Development

### 5.1. Metallothionein Expression in P. sicula Embryos under Natural Conditions

Despite it being demonstrated that null-MT mice are viable and reproduced normally [48], under natural conditions *MT* genes are precociously expressed in embryos, thus reinforcing the idea that MTs play critical functions during embryonic development. In mammals, MT transcripts are present as maternal messengers stored in eggs, and the embryonic *MT* genes are among the first to be transcribed, first in blastocyst and then in organs such as the liver and kidney [49,50].

As described above, in *P. sicula* eggs, MT mRNA accumulates abundantly; as the next step, we decided to investigate by in situ hybridisation the spatiotemporal changes in transcript localisation throughout development, from egg deposition to hatching (about 50 days later). Freshly laid eggs were incubated in a terrarium maintained at natural conditions of humidity and temperature, and embryos collected at regular time intervals [31].

In *Podarcis* blastula, collected when the fertilised eggs are still in oviducts, MT transcripts of maternal origin are present in both embryonic and extra-embryonic areas.

**Figure 5:**
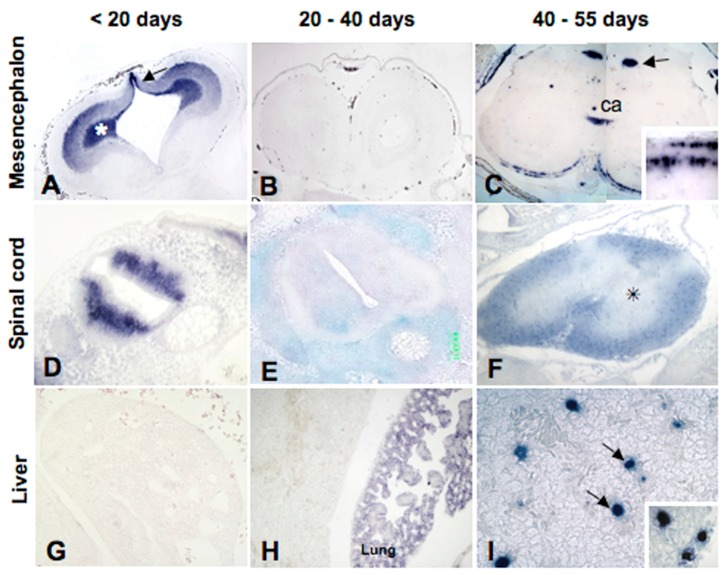
Changes in MT mRNA localisation during *P. sicula* development. Photographs are modified from [31]. (**A**–**C**) Cross-section of the optic lobes at different stages. (**A**) Mesencephalon at 20 days post deposition (pd): MT mRNA labeling is concentrated in the developing cortex (*); the dorsal ventricular zone is intensely labeled (arrow); (**B**) At 40 days pd, mesencephalon is completely unlabeled; (**C**) In the pre-hatching embryo the transcript is on the cortex (insert), commissura ansulata (ca) and in the basal nuclei (arrow). (**D**–**F**) Cross-section of spinal cord. (**D**) Neural tube at 5 days pd, with positive cells concentrated in the developing grey matter; (**E**) Unlabeled neural tube; (**F**) Neural tube with unlabeled grey matter (*) and labeled white matter; (**G**–**I**) Cross-section of liver. Messenger is absent at 10 (**G**) and 40 days pd (**H**); parenchymal lung cells are labeled at 40 days. In the pre-hatching embryos (**I**) Kupffer cells are positive (arrows, insert). Objectives: (**A**–**F**): 10×; (**G**–**I**): 20×; (inserts **C**,**I**): 40×.

Later, at the beginning of organogenesis (5–10 days post deposition (pd)), MT transcripts are present in the cytoplasm of the developing neurons of the central nervous system and in the undifferentiated retina. Transcripts are also present in mesodermal derivates such as somites and mesenchyme, but not in the kidney or in endodermal derivates like the liver and gut (Figure 5).

At 20 days from deposition, the MT-mRNA distribution slightly changes, and from this moment on the embryonic gene is expressed, at least in some regions. In the brain, for example, where the typical anatomical districts are recognisable, transcripts are present in the ventricular zones of telencephalon and diencephalon, in the developing optic cortex and in several nuclei of mesencephalon and medulla oblongata, in the ependymal cells of all vesicles, and in the horns of the spinal cord. In the retina, the MT mRNA is present in the ganglion cells and in the inner and outer nuclear layers’ cells. In the trunk, only the renal glomeruli show the presence of the MT transcript, whereas in the renal tubules, and lung, liver, and gut mucosa they are still absent (Figure 5).

At 40 days pd, MT transcripts simultaneously disappear from the telencephalon, diencephalon, mesencephalon, and ganglion layers of the differentiating retina and the grey matter of the spinal cord. However, they remain in the nuclei of the medulla oblongata, in the granular and molecular layers of the cerebellum, and appear, for the first time, in the white matter of the spinal cord. This shift in MT localisation in the central nervous system could be explained by a consumption of the pool of maternal mRNAs and/or a temporary silencing of the MT embryonic gene. In the trunk region, the MT mRNA is present in the renal glomeruli and for the first time in the lung parenchyma, whereas it is still absent in the liver and gut mucosa and the kidney tubules (Figure 5).

Immediately before hatching, at 50–55 days pd, the distribution of MT transcripts undergoes further relevant changes. Regarding the central nervous system, they appear in the cortical areas of the telencephalon and mesencephalon, in several basal nuclei, and in ependymal cells. Their distribution in the medulla oblongata and in the spinal cord does not change with respect to the previous stage. In the fully differentiated retina, the MT mRNA is present in the outer and inner nuclear layers, and again in the ganglion cells layer. In the other organs, transcripts are observed for the first time in the gut mucosal cells and in the liver, but not yet in the kidney tubules (Figure 5).

Taken together, these results show a complex modulation of *MT* gene expression during lizard development, along with several differences when compared with mammalian embryos, in which the expression of *MT* gene is activated early and abundantly in the visceral organs with detoxifying functions, whereas the gene is activated late, at birth, in the brain [51].

### 5.2. Metallothionein Expression in P. sicula Embryos Incubated in Cadmium-Contaminated Soil

The absence of MT transcripts in the gut, liver, and kidney of *Podarcis* embryos might be correlated to the peculiarities of their development: indeed, as all terrestrial oviparous vertebrates, they develop and grow using the nutrients inside the eggs, and detoxifying organs are not yet required.

Many studies indicate that MTs protect embryos from intracellular damage caused by harmful substances, particularly heavy metals [52]. Embryo potential exposure and uptake to toxic heavy metals, such as cadmium, varies greatly: in mammals, the placenta is the primary target [53], whereas in oviparous tetrapods, vitellogenesis is considered the primary route of accumulation of toxic metals in eggs [54]. However, it has been demonstrated that metals and other soluble organic contaminants are able to cross the breathable eggshell of many reptiles, affecting embryos’ morphology and gene expression [55,56,57].

**Figure 6:**
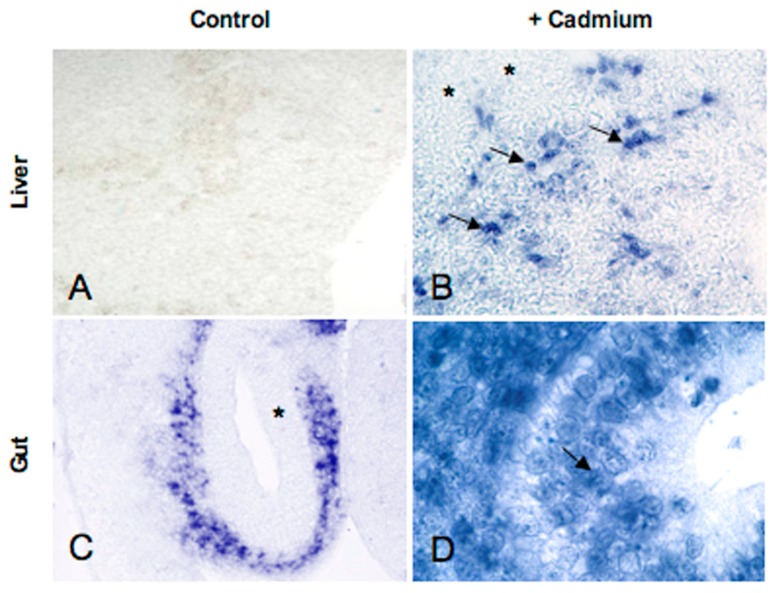
Cd-induction of MT expression in the liver and gut of a *P. sicula* embryo. Photographs are modified from [58]. (**A**,**C**) Control embryo at 10 days pd. (**A**) Unlabeled liver parenchyma; (**C**) gut mucosa completely unlabeled (*). (**B**,**D**) Cd-treated embryo at 10 days pd. (**B**) Several sinusoidal cells (arrows) are labeled, liver parenchyma () is unlabeled; (**D**) gut mucosal cells show intensely labeled nuclei (arrow). Objectives: (**A**,**B**): 20×; (**C**): 10×; (**D**): 40×.

So, we decided to evaluate the spatiotemporal changes in MT expression in embryos incubated in Cd contaminated soil (50 mg Cd/Kg soil) [58]. A preliminary histological analysis demonstrated no embryo mortality, but the onset of severe morphological alterations mostly concentrated in the encephalic and optic areas; no alterations were observed in the medulla oblongata, spinal cord and visceral organs throughout development. Interestingly, in situ hybridisation analyses demonstrate a Cd-induced spatiotemporal shift in embryonic *MT* gene expression in some trunk organs but not in the developing brain and retina. In particular, MT transcripts were present in the intestinal mucosa and in the liver sinusoids in 10-day-old embryos (Figure 6), whereas in the control embryos MT mRNA appears only immediately before hatching.

These results support the hypothesis that the MT mRNA is absent in detoxifying organs, since they are not functional during development; however, if solicited by toxic substances, the gene is activated and transcription starts. On the other hand, the morphological alterations observed demonstrate that the cephalic area of Cd-treated embryos is exposed to the toxic effects of cadmium. At this point it is necessary to determine whether the transcripts are translated or, alternatively, whether proteins translated from these transcripts are engaged in functions other than heavy metal detoxification.

## 6. Conclusions

In adult lizard *Podarcis sicula* the MT protein is present in almost all tissues, as expected considering its fundamental role in modulating intracellular zinc/copper concentrations. Exogenous metals, and cadmium in particular, may trigger a significant upregulation, but the timing and amount of newly synthesised mRNA and/or protein is clearly dose- and tissue- specific. Further analyses are needed to draw conclusions on the relevance of measuring the MT transcripts in some tissues of *P. sicula* to reveal Cd contamination, and to determine if the proteins translated from these transcripts are engaged in functions other than heavy metal detoxification. The particularly complex pattern observed in the tissue distribution and the response to cadmium exposure suggests that MTs might have many different roles, some probably still to be discovered. The elusive MT therefore is far from being known in full, especially in this reptile.

## Figures and Tables

**Table 1:** Abundance of metallothionein transcript and protein in *P. sicula* tissues, under natural conditions and after in vivo Cd-exposure.

Tissue	Control	Acute Cd-Exposure	Chronic Cd-Exposure
	mRNA/Protein	mRNA/Protein	mRNA/Protein
Brain	+++/n.d.	No change/n.d.	No change/n.d.
Liver	++/+++	+3-fold/+2-fold	No change/+2-fold
Kidney	++/n.d.	+4-fold/n.d.	+4-fold/n.d.
Ovary	+/not present	+3-fold/+++	+4-fold/++
Gut	+/n.d.	n.d./n.d.	+30-fold/n.d.

n.d. = not determined. MT relative abundance : + < ++ < +++.
